# Molecular Aspects of Dendritic Cell Activation in Leishmaniasis: An Immunobiological View

**DOI:** 10.3389/fimmu.2019.00227

**Published:** 2019-02-22

**Authors:** Rafael Tibúrcio, Sara Nunes, Ivanéia Nunes, Mariana Rosa Ampuero, Icaro Bonyek Silva, Reinan Lima, Natalia Machado Tavares, Cláudia Brodskyn

**Affiliations:** ^1^Gonçalo Moniz Institute, Oswaldo Cruz Foundation, Salvador, Brazil; ^2^Federal University of Bahia, Salvador, Brazil; ^3^Instituto Nacional de Ciência e Tecnologia (INCT) iii Instituto de Investigação em Imunologia, São Paulo, Brazil

**Keywords:** dendritic cell activation, *leishmania*- dendritic cell interaction, parasite uptake, dendritic cells migration, metabolism of infection, epigenetic modifications

## Abstract

Dendritic cells (DC) are a diverse group of leukocytes responsible for bridging innate and adaptive immunity. Despite their functional versatility, DCs exist primarily in two basic functional states: immature and mature. A large body of evidence suggests that upon interactions with pathogens, DCs undergo intricate cellular processes that culminate in their activation, which is paramount to the orchestration of effective immune responses against *Leishmani*a parasites. Herein we offer a concise review of the emerging hallmarks of DCs activation in leishmaniasis as well as a comprehensive discussion of the following underlying molecular events: DC-*Leishmania* interaction, antigen uptake, costimulatory molecule expression, parasite ability to affect DC migration, antigen presentation, metabolic reprogramming, and epigenetic alterations.

## Introduction

### Important Considerations in Leishmaniasis

Leishmaniasis comprises a collection of neglected protozoan infections caused by unicellular organisms belonging to the genus *Leishmania* spp. According to the current World Health Organization estimation, 12 million people are affected by leishmaniasis and 350 million are at risk of infection worldwide (1–3).

The pathology of this disease results in a wide spectrum of clinical manifestations not only associated with the biological aspects of *Leishmania* species and strains, but also with host immune responses. Interestingly, it has been recently suggested that the clinical progression of the disease is influenced by several other factors, ranging from the host's nutritional status to the presence of RNA viruses in the *Leishmania* species (4–7).

These manifestations are dichotomically divided into Visceral (VL) and Tegumentary Leishmaniasis (TL). The former is characterized by the dissemination of parasites to visceral organs, while the latter branch includes Localized Cutaneous Leishmaniasis (LCL), a frequent form of TL in which ulcerated skin lesions are common. It has been abundantly reported that a modest fraction of LCL cases can evolve into mucosal lesions, which is termed as Mucocutaneous Leishmaniasis (MCL). Additionally, TL can also present as a variety of clinical manifestations, such as Disseminated Cutaneous Leishmaniasis (DCL), which comprises multiple nodular ulcerated lesions, whereas Diffuse Leishmaniasis (DL) is characterized by scattered non-ulcerated lesions (5, 8, 9).

*Leishmania* transmission occurs when infected sandflies inoculate the promastigote forms of the parasite into the host skin. Additionally, the arthropod vector also introjects various parasite-associated compounds, along with other molecules found in salivary secretions, which collectively exert immunomodulatory effects on the host defense (10). The early events of infection are characterized by the engagement of different phagocytic cells (e.g., tissue-resident macrophages, dermal DCs, and neutrophils) in the recognition and uptake of parasites (8). Emerging pieces of evidence indicate that neutrophils are one of the first cell types to interact with *Leishmania* parasites (11). Subsequently, depending chiefly on the *Leishmania* species, infected neutrophils become apoptotic and can be phagocytized by macrophages (12). Accordingly, parasite transmission to these cells becomes facilitated, leading to the subsequent differentiation of promastigotes into intracellular replicative amastigotes that occurs in the interior of macrophages phagolysosomes. Additionally, the literature upholds that dendritic cells (DCs) are also key elements in the early interaction with *Leishmania* parasites, thusly these are thought to be a decisive in the outcome of infection (13). Indeed, the complex interactions occurring between DCs and parasites may lead to long-term *Leishmania* replication, or to the establishment of an effective immune response against this pathogen.

### The Immunobiology of Dendritic Cells

DCs are competent antigen presenting cells (APC) that take center stage in both the induction of immunological responses and the generation of tolerance (14). In the context of inflammation and infection, DCs are responsible for orchestrating the connection between the innate and adaptive axis of immunity. Interestingly, despite the significant importance of DCs in several immunological processes, these cells do not comprise a homogeneous population, and are further classified into distinct subtypes according to origin, differential expression of surface proteins, cell localization, and immunological function (15).

#### Dendritic Cell Origin

It has been long hypothesized that DCs stem from a bone-marrow resident population of hematopoietic stem cells (HSC), which eventually give rise to both granulocyte-macrophage progenitors (GMP), and multi-lymphoid progenitors (MLP), the precursors of all DC subsets (16). Subsequent stages in DC ontogeny involve precursors, such as CD14+ monocytes, circulating blood myeloid DCs (mDCs), or plasmacytoid DCs, from which all myeloid and lymphoid DCs are derived. It is noteworthy that mDC precursors comprise a heterogeneous lineage of cells predetermined to develop into CD1+ or CD141+ DCs. Additionally, human mDCs express conventional myeloid markers, including CD11c, CD11b, CD13, and CD33 (17). In mice, these cell populations are often referred to as conventional DCs. Interestingly, it has been well-elucidated that in humans, both CD14+DCs and inflammatory DCs are derived from classical monocytes, which justifies the fact that these cells present greater similarity to monocytes and macrophages than other DC subsets (18).

#### Dendritic Cell Subtypes

##### Myeloid/Conventional Dendritic Cells

Typically, myeloid DCs are classified into two subtypes: cDC1 and cDC2. The human cDC1 subset is identified by the expression of CD141 (BDCA-3), while the murine equivalent is subdivided into a splenic CD8α-bearing population and another CD141+ DC subset residing in non-lymphoid tissues (19–21). Human and mouse cDC1 express Clec9A (C-type lectin domain family 9-member A) and XCR1 (a chemokine receptor), which provide specificity for their biological activities in combating invasive microorganisms and tumors (22). In regard to the expression of transcription factors, cDC1s are characterized as producing both BAFT3 (Basic leucine zipper transcription factor) and IRF8 (Interferon regulatory factor 8). It has been long suggested that cDC1s have the capacity to effectively induce the activation of CD8+ T cells via the process of antigen cross-presentation, as well as produce copious amounts of IL-12p70 (23, 24).

**Figure 1:**
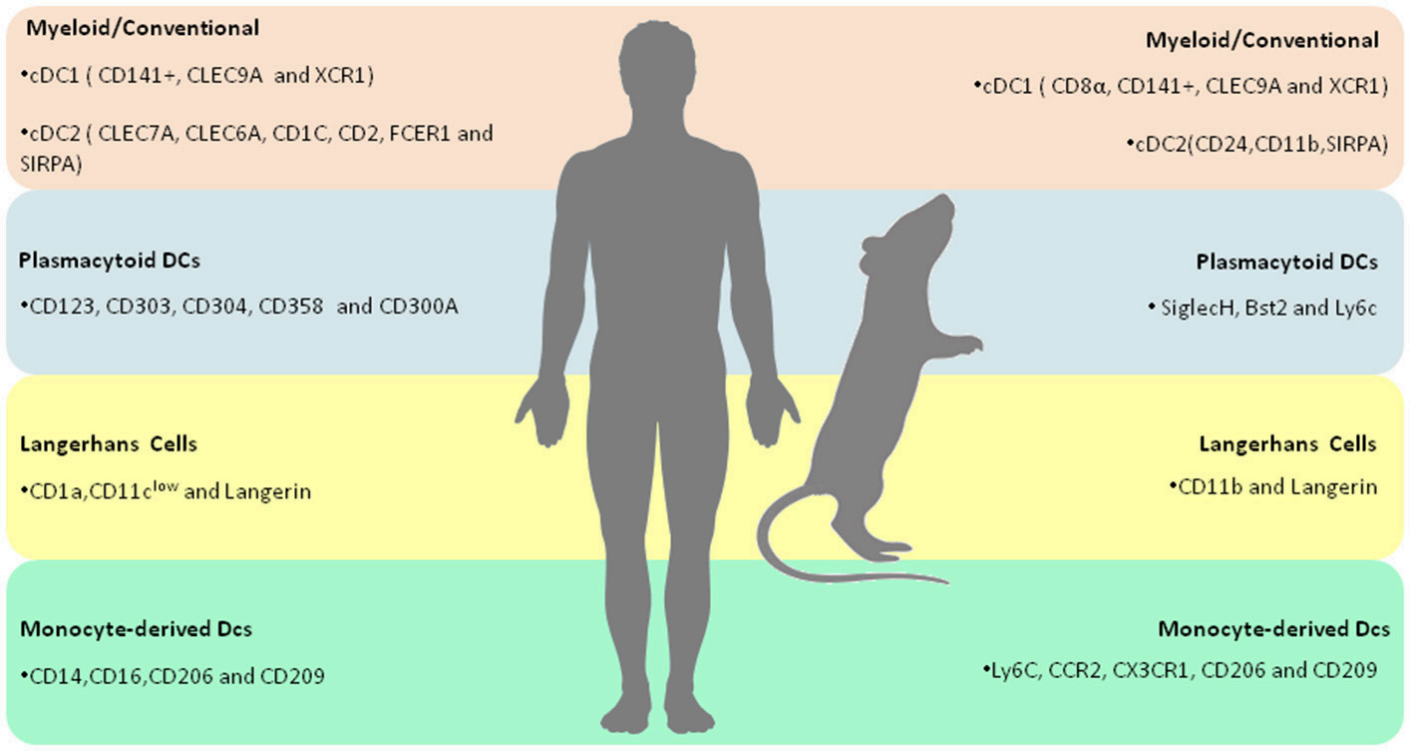
Overview of human and murine DC subtypes–Summary of the molecular markers that characterize each human and murine DC subtypes.

cDC2s express both common myeloid markers, such as CD11b, CD11c, CD13, and CD33, in addition to other antigens more recently identified in these cells: CD1c, CD2, FceR1, and SIRPA (15). cDC2s comprise a large portion of the human conventional DCs found in blood and tissues. The immunological function of cDC2s is granted by a myriad of immune receptors, including Toll-like receptors (TLRs) 2,4,5,7 and 8, C-type lectins, including Dectin-1 and−2 as well as Nod-and RIG-like receptors (Figure 1 and Table 1) (15).

**Table 1:** Summary of the hallmarks of *Leishmania* induced DC activation.

**Leishmania-prompted effects in DC activation**	**DC subsets**	**Parasite species**	**Experimental model**	**Functional aftermath**	**References**
TLR9 activation	BMDCs	*L. infantum*	C57BL/6	Increases Neutrophil chemoattraction and IL-12 production	Sacramento et al. (53)
		*L. major*		Enhances IFN-γ production and cytotoxicity in NK cells	Liese et al. (54)
TLR2/TLR4 activation	BMDCs	*L major*	BALB/c	Upregulation of CD80, CD86, and MHC-II expression	Komai-Koma et al. (49)
TLR2 activation	BMDCs	*L. braziliensis*	C57BL/6	Decreases DC activation and IL-12 production	Vargas-Inchaustegui et al. (50)
Engagement of MyD88 signaling pathway	BMDCs	*L. braziliensis*	C57BL/6	Enhances IL-12 production	Vargas-Inchaustegui et al. (50)
A2B receptor activation	BMDCs	*L amazonensis*	C57BL/6J	Decreases of CD40 expression and IL-12 production	Figueiredo et al. (64)
Increased expression of HIF1 α	Murine splenic DCs	*Leishmania donovani*		Decreases production of IL-12, parasite survival, limited generation of Th1 cells	Hammami et al. (111)
Up-regulation of MHC class II, CD40, CD54, CD80, and CD86	Epidermal Langerhans cells-like DC	*L. major* amastigotes	C57BL/6	Increase production of IL-12	Von Stebut et al. (78)
Did not change the expression of CD80, CD54, and MHC II molecules	BMDCs	*L. mexicana* amastigotes	C57BL/6	Did not alter the production of IL-12	Bennett et al. (79)
Lower levels of MHCII, CD86, and CD40 expression	BMDCs	*L. amazonensis*	C57BL/6	Declined T-cell proliferation	Figueiredo et al. (65)
Low levels of CD40 expression	BMDCs	*L. donovani*	BALB/c	T regulatory cells expansion and disease intensification	Martin et al. (71)
Fail in producing IL-12 through a CD40-dependent manner	BMDCs	*L. amazonensis*	BALB/c	Increase in IL-4 levels	Qi et al. (74)
Down-regulation of CD80 and up-regulation of CD86	Human moDCs	*L. amazonensis*	*in vitro*	Increase in IL-6 during DC differentiation	Favali et al. (75)
Alterations in DC migration		*L. major*		Inhibition of DCs motility	Steigerwald et al. (98); Ponte-Sucre et al. (100)

*BMDC, bone marrow -derived dendritic cell; IFN-γ, interferon gamma; IL-12, interleukine-12; NK, natural killer cells; TLR, toll-like receptors; HIF1α, hypoxia-inducible factor 1 α*.

##### Plasmacytoid Dendritic Cells

Plasmacytoid DCs (pDCs) comprise a group of Type I interferon (IFN)-producing cells whose distinguishing feature is their participation in the response against viral infection. Interestingly, human pDCs were first identified as a population of cells found in the peripheral blood and tonsils. With respect to morphology, blood pDCs are mainly recognized by their resemblance to lymphocytes, whereas IL-3/CD40L-cultured pDCs possess a microscopic appearance similar to mDCs (22, 25). Typically, human pDCs are characterized by the unique expression of both cell-surface receptors and transcription factors. Of note, the distinctly expressed receptors include both conventional and other recently identified markers. The former includes CD123 (IL-3R), CD303 (BDCA-2), and CD304 (BDCA-4), while the later includes FceR1, DR6 (CD358), and CD300A. As previously mentioned, these cells also exhibit distinctive TF expression, including E2-2, IRF8, and IRF4 (26, 27). It is particularly interesting to note that, in contrast to cDCs, human pDCs do not express any conventional myeloid markers, e.g., CD11b, CD11c, CD13, and CD33 (15). Murine pDCs express siglecH (Sialic acid-binding immunoglobulin-type lectin), bst2 and Ly6C. SiglecH is a surface receptor responsible for binding glycans presenting sialic acids residues. Interestingly, Blasius et al demonstrated that siglecH regulates type I IFN production in a DAP12-dependent manner (28). Bst2, an integral membrane protein associated with lipid rafts, has also been associated with IFN-mediated responses against viral infection (29). Additionally, several reports have demonstrated that both murine and human pDCs rely on TLR7 and TLR9 expression for IFN-α production and immunity against viruses (30). Interestingly, mounting evidence have demonstrated that pDCs also play a role in the establishment of peripheral tolerance by delivering antigens to the lymph nodes (Figure 1 and Table 1) (31–33).

##### Langerhans Cells

A distinct lineage of epidermis-resident DCs, known as Langerhans cells (LC), mainly characterized by the expression of C-type lectin Langerin and CD1a, grant organisms immunity against several skin pathogens, such as fungi and bacteria (34). Uniquely, LCs possess Langerin-replete organelles, known as Birbeck granules. Although their main function has not been well-elucidated, the depletion of these granules has not been determined to mitigate the process of antigen presentation (Figure 1 and Table 1) (35).

##### Monocyte-Derived Dendritic Cells

Monocytes constitute a very plastic group of mononuclear phagocytic cells long thought to be the source of macrophages and DCs. Several reports suggest that monocytes possess both pro- and anti-inflammatory functional specializations which are, in turn, chiefly regulated by tissue environments (36). Human monocytes comprise two subsets of peripheral blood circulating cells mainly characterized by the expression of CD14 and CD16, whereas murine monocytes can be identified by the presence of Ly6C, CCR2, and CX3CR1 (22). Of note, in the presence of inflammation, blood-circulating monocytes invade tissues, subsequently differentiating into monocyte-derived Dendritic cells (moDCs). It has been long established that *in vitro* moDCs are obtained by stimulating monocytes with GM-CSF and IL-4 (37). These cells possess a broad functional repertoire, including lymphocyte activation and the production of cytokines, such as IL-6, TNF-α, IL-12, IL-23, and IL-1 (15). Inflammatory DCs (iDCs) express both CD14 and CD16, in addition to CD206, CD209 (DC-SIGN), and CD163. Importantly, a novel tumor necrosis factor (TNF)-and inducible nitric oxide synthase (iNOS)-producing DCs (TIP-DCs) subset has been reported to exert a pivotal role in the course of several infectious diseases, including experimental leishmaniasis (Figure 1 and Table 1) (38).

##### Adaptative Immunity Gatekeepers: The Role of DCs and T Cells Activation

In an immature state, DCs are typically located in peripheral tissues and express low levels of major histocompatibility complex II (MHC II) and costimulatory molecules. These cells possess highly efficient cellular machinery for antigen recognition and capture (39). In response to signals associated with infection and inflammation, such as the presence of pathogens and other damaging elements, DCs undergo intricate molecular processes that culminate in the acquisition of a mature functional state, whose main characteristic is the ability to induce both naïve CD4^+^ T cell activation and proliferation via antigen presentation (40). Most importantly, the signaling process that induces DC maturation involves the recognition of pathogen-associated molecular patterns (PAMPs) by way of a sophisticated surface and intracellular molecular detection system consisting of pattern recognition receptors (PRRs) and downstream signaling (41).

After interaction with antigen-bearing DCs, naïve CD4^+^ T cells are capable of differentiating into two main functional phenotypes: T helper 1 (Th1) and T helper 2 (Th2) profiles. It should be noted that this dichotomy is a rather simplistic representation of the Th cell repertoire. In recent decades, several studies have identified other Th subtypes, including Th17 (whose hallmark is the production of IL-17 in response to viruses, bacteria and fungi), Th9 (a producer of IL-9 and IL-10, and also a key element in humoral interplay with B cells), Th follicular (characterized by the production of IL-4 and IL-2 (also related to supporting B cell-mediated immunity), and T regulatory (Tregs), involved in the promotion of self-tolerance (42). Since T regs exert a prevalent immunological role in the regulation of other immune cells, their populations heterogeneity and functional specializations are of particular interest. Commonly, T regs are dichotomically classified as “natural” (CD4^+^CD25^+^Foxp3^+^ T cells) or inducible T regs (a group that includes the IL-10-secreting Treg1 cells, the Th3 population that produces both TGF-b and IL-10, and foxp3^+^ Tregs). A more detailed description of the diversity and functions of T regulatory cells can be found elsewhere (43).

##### DC as Modulators of the Adaptive Immune Response in Leishmaniasis

The biological features of pathogens and activation PRRs as well as the underlying signaling processes, are determinant in the specific cytokines secreted by activated DCs, which in turn are one of the key element in the polarization of Th cell subtypes (44).

In general, Th1 cells produce pro-inflammatory cytokines, such as Interferon Gamma (IFN-γ), which lead to Tumor Necrosis Factor Alpha (TNF-α) production by innate immune cells, promoting a resistance profile against *Leishmania*. The hallmark of IFN-γ leishmanicidal activity relies on the classical activation of infected macrophages, leading to increased production of nitric oxide (NO) and reactive oxygen species (ROS), which subsequently culminate in intracellular *Leishmania* elimination (13). By contrast, the Th2 profile is characterized by the production of IL-4, IL-5, and IL-13, which are mostly associated with enhanced arginase activity accompanied by the alternative activation of macrophages, parasite survival and proliferation, and pronounced susceptibility (13). Additionally, in recent decades, the contributions of the Th17 subtype on the progression of leishmaniasis has become a growing concern. The hallmark of the Th17 profile is the production of IL-17, and the subsequent recruitment of neutrophils to the site of inflammation. As reviewed elsewhere, the joint actions of this subtype paradoxically play a dual role in leishmaniasis, since these cells are not only responsible for the elimination of parasites, but also for the exacerbation of the inflammatory process and tissue damage (45). Crosstalk between *Leishmania* and DCs via the stimulation of various cellular apparatuses and the engagement of multiple signaling processes culminates in phenotypic and functional alterations in DCs. Such modifications are imperative for proper cytokine production and the activation of Th cells, which induce immune events that can result in parasite control (46).

## Dendritic Cell-*Leishmania* Interaction

### Leishmanial Signals Prompt DCs Activation

One of the biological functions of DCs is to recognize molecular patterns associated with pathogens (PAMPs). To this end, DCs employ PRRs that interact with a variety of PAMPs expressed by distinct species of *Leishmania*. The activation of DCs can be substantially modulated by these interactions, which greatly influence the immune response against *Leishmania* (46).

#### The DC Recognition Apparatus: the Role of PRRs in Leishmania-DC Interplay

Toll-like receptors (TLRs) are germline-encoded immune receptors that play a pivotal role in the immunosurveillance function of DCs. These receptors are subdivided into 10 families in humans (TLR1 to TLR10) and 12 families in mice (TLR1 to TLR9, and TLR11 to TLR13) (47), and are expressed on either the cell membrane surface or in intracellular compartments. TLRs possess leucine-rich repeats that serve as molecular docking sites for ligand-receptor interactions. Upon ligand-mediated activation, TLRs undergo an intricate dimerization process that activates a variety of biochemical pathways, culminating in the transcription of several inflammatory genes. Of note, it has been proposed that TLRs may be central elements in the establishment of immune homeostasis, as these cells participate in the delicate balance between pro-inflammatory and anti-inflammatory responses (48). However, despite their relevance in the recognition of several pathogens and the induction of immune responses, only TLR2, TLR4, and TLR9 have been described in the mediation of DC-*Leishmania* interaction.

It has been demonstrated that the neutralization of TLR2 and TLR4 decreases the expression of molecules involved in the process of antigen presentation during *L. major* infection, which suggests that both receptors may be key players in the establishment of effective responses against *Leishmania* (49). Interestingly, a study documented that TLR2 deficiency increases DC activation, leading to IL-12 production during *L. braziliensis* infection. On the other hand, a deficiency of MyD88 results in lower levels of DC activation and IL-12 production, both essential elements in mounting protective immunity against *L. braziliensis* (50).

TLR2 also recognizes Lipophosphoglycan (LPG), a surface molecule conserved in all *Leishmania* species that is considered an important virulence factor, especially due to its role in the modulation of immune cell activation (51). When LPG of *L. mexicana* is recognized by the TLR2 of moDCs, the expression of MHC-II and CD86 as well as the secretion of IL-12p70, are enhanced. Subsequently, the interaction of DCs with NKT cells culminates in higher IFN-γ production. This cellular interaction could possibly contribute to the protective state observed during the acute phase of *L. mexicana* infection (52).

TLR9 has been described as important in DC activation as well as in the production of neutrophil chemoattractant during *L. infantum* infection in C57BL/6 mice (53). Additionally, TLR9 is also required for the induction of IL-12 production in mouse bone marrow-derived DCs (BMDCs) infected with *L. major*, leading to both IFN-γ expression and cytotoxicity enhancement in NK cells (54). Collectively, these findings contribute to the understanding of how the intracellular TLRs in DCs mediate the stimulation of other immune cells that promote parasite eradication.

Different *Leishmania* species can mitigate the signaling pathways of CTLRs to promote parasite proliferation and survival. Iborra et al. showed that *L. major* releases a soluble protein ligand of Mincle (Macrophage inducible Ca^2+^-dependent lectin receptor) that targets an inhibitory ITAM signaling pathway, resulting in the impairment of DC activation and migration (55). In a similar vein, Zimara et al. experimentally demonstrated the importance of CTLRs in mounting an adaptive immune response against *L. major*. These researchers observed an increased expansion of Dectin-1^+^ DCs following *L. major* inoculation in C57BL/6 and BALB/c mice as well as in the peripheral blood of CL patients. Additionally, experiments with BMDM stimulated with a Dectin-1 agonist revealed both high levels of DC maturation and the expansion of CD 4 T cells (56). Collectively, these findings serve to indicate the significance of both CTRL signaling and the physical interactions between these cells and pathogens in the promotion of an effective immune response.

#### Mechanisms of Leishmania Uptake

In addition to their importance as major mediators of the innate and adaptive branches of immunity, DCs are also recognized for their highly efficient phagocytic activity (57). These cells actively collect antigens in their surroundings, and couple subsequent antigen processing with epitope exhibition via antigen presentation platforms–the major histocompatibility complex molecules (58).

Typically, the mechanisms of capturing pathogens involve specific receptor-ligand interactions as well as the mobilization of cytoskeleton elements that promotes the internalization of parasites (59). Several studies have proposed that the uptake of *Leishmania* by DCs occurs in a parasite life form-dependent manner, since DCs preferentially phagocytose IgG -coated amastigotes. In fact, amastigotes internalization involves the participation of FcγRI and FcγRIII (60). It has been suggested that GP63, a protease found on leishmanial membranes, mediates the conversion of C3b into its inactive form, iC3b. Subsequently, iC3b binds to CR3, resulting in the adherence of leishmania to the surface membrane of DCs (60).

Argueta-Donohué et al. demonstrated that DC-SIGN (Dendritic Cell-Specific Intercellular adhesion molecule-3-Grabbing Non-integrin), a surface receptor mainly found in DCs, mediates a more efficient internalization rates of *L. mexicana* promastigotes after 3 h of *in vitro* infection (52). Moreover, these authors also confirmed that the experimental neutralization of DC-SIGN significantly reduces the rates of infection in moDCs. These intriguing findings illustrate the fundamental role of DC-SIGN in several instances of *Leishmania-*DC interplay, ranging from the initiation of parasite phagocytosis to discrimination between *Leishmania* life cycle stages (52). Additionally, mounting evidence indicates that DC-SIGN also recognizes *L*. *pifanoi* surface molecules, contributing to the subsequent uptake of parasite amastigotes (61). Furthermore, *L. major* and *L. donovani-* infected moDCs exhibit reduced surface expression of DC-SIGN in contrast to uninfected cells, with this immunomodulation being accentuated in cells stimulated with excreted-secreted antigens (ESA) of both *Leishmania* species (62). Together, these studies suggest that the consequences of the DC-SIGN-mediated crosstalk between *Leishmania* and host DCs may have profound biological consequences in *Leishmania* infection.

#### The Effects of Purinergic Receptors on DC Activation During Leishmania Infection

Purinergic receptors play a significant role in the recognition of damage-associated molecular pattern (DAMPs), including the detection of extracellular Adenosine Triphosphate (ATP), a potent pro-inflammatory trigger of immune responses (63). In pathophysiological contexts, ATP is converted into Adenosine (ADO) via the action of the ectonucleotidases CD39 and CD73, and the accumulation of ADO in the extracellular milieu results in the activation of its A2 receptor. This phenomenon has been observed during *L. amazonensis* infection, which was accompanied by the suppression of DC functions via decreased rates of CD40 expression and IL-12 production. Additionally, the activation of the A2b receptor of DCs decreases the capacity of these cells to stimulate T cell proliferation and the production of IFN-γ, leading to an insufficient protective immune response, a peculiarity of *L. amazonensis* infection (64). While increases in CD39 and CD73 expression are also observed in *L. braziliensis* and *L. major* infection, A2b receptor activation has not been detected. Interestingly, it has been proposed that the main evasion mechanism employed by these two species is reduced expression of the molecules involved in antigen presentation, which includes the exploitation of the IL-10 receptor (IL-10R). Notwithstanding, this evasion mechanism employed by *L. braziliensis* and *L. major* is followed by the upregulation of CD40, which may suggest that it does not prevent T cell activation (65).

Together, these findings provide evidence that early interactions between DCs and *Leishmania* can have profound effects on disease outcome. Several of the mechanisms of immune evasion employed by *Leishmania* include the mitigation of DC immunobiological functioning via the exploitation of different receptors and the disruption of downstream signaling pathways. In addition, recent data indicate that the impairment of DC activation is directly associated with the enhancement of parasite survival and persistence in hosts.

## Co-stimulatory Molecules And Antigen Presentation

Following the recognition and internalization of pathogens, DCs migrate to secondary lymphoid organs to present processed antigens to naïve T cells (66). Subsequently, the adaptive immune response becomes initiated via the presentation of small peptides through either MHC class I or class II molecules. Basically, the former class mediates the recognition of endogenous peptides by cytotoxic CD8+ T cells, while the latter is involved in the presentation of exogenous peptides to CD4+ T helper cells. Notably, the process of antigen fragmentation is of paramount importance to allow for proper antigen presentation, since MHCII molecules only present peptides with a specific number of amino acids (67). Alternatively, DCs are also capable of mobilizing MHC I molecules in order to display exogenously derived-antigens, a process known as cross-presentation (68). Additionally, co-stimulatory molecules (such as CD40, CD80, and CD86) are essential to effective antigen presentation, by providing secondary signals for T cell expansion and differentiation (69).

Several species of *Leishmania* employ distinct strategies to regulate the expression of co-stimulatory molecules, which dampens the process of antigen presentation (70). Accordingly, the modulation of co-stimulatory molecules can be associated not only with enhanced parasites survival and growth, but also with subsequent disease outcome.

Figueiredo et al. experimentally demonstrated that *L. amazonensis* induced lower levels of MHCII, CD86 and CD40 expression in BMDCs (bone marrow -derived DCs) from C57BL/6 mice, resulting in a decline in T-cell proliferation (65). Furthermore, the adoptive transfer of BMDCs expressing low levels of CD40 was associated not only with T regulatory cell expansion, but also with an increase in *L. donovani* burden in BALB/c mice (71). It has been also shown that CD40 and its ligand are important for the development of resistance against *L. major* infection (72, 73). Hai Qi et al. reported that *L. amazonensis* amastigotes mitigated IL-12 production in a CD40-dependent manner in a BALB/c infection model, which was followed by an increase in levels of IL-4 (74). Subsequently, amastigote-infected DCs were observed to be able to activate pathogenic CD4+ T cells, which could potentially lead to exacerbated *Leishmania* proliferation and the progression of pathogenesis (72). Thus, emerging evidence suggests that reduced CD40 expression could possibly facilitate *Leishmania* infection.

The importance of CD80 and CD86 expression has been highlighted in the establishment of early immune responses. For instance, the infection of human moDCs with *L. amazonensis* downregulates the expression of CD80 and upregulates the expression of CD86, which is followed by a decrease in IL-6 production during DC differentiation (75). Although CD86 possibly takes center stage in this context, the equivalent expression of other costimulatory molecules can lead to the early production of IFN-γ or IL-4 during infection by *L. major* depending on the experimental model (76). Together, these results reinforce the contribution of these molecules in the production of different cytokines by properly stimulated T cells.

Several species of *Leishmania* can modulate antigen processing and the expression of MHC II molecules (77). DCs infected with *L. major* amastigotes not only upregulate the expression of several molecules involved in antigen presentation, such as MHC class II, CD40, CD54, CD80, and CD86, but also exhibit elevated rates of IL-12 production. It should be noted that only the amastigote forms of these parasites were capable of inducing this increase in *L. major*-infected DCs (78). Conversely, *L. mexicana* amastigotes do not promote increased expression of CD80, CD54, and MHC II molecules in BMDCs, suggesting that these discrepancies in the immune response by DCs occur in a species-specific manner (79).

Interestingly, a recent study by Resende et al. reported a dichotomic response between *L. infantum*-infected and non-infected DCs. In this study, the authors observed that uninfected DCs expressed higher levels of IL-12p40 and other co-stimulatory molecules, which enabled DCs to elicit appropriate CD4^+^ T cell immunoprotective responses, whereas infected DCs expressed lower levels of co-stimulatory molecules and high IL-10 production (80). This finding suggests that *L.infantum*-infected DCs and their uninfected counterparts exert antagonistic roles in the activation and polarization of T cells, mechanistically revealing a novel evasion strategy employed by this species. Along the same lines, a study carried out by Carvalho et al demonstrated that, in contrast to *L-braziliensis*-infected DCs, only uninfected DCs upregulate the expression of MHC II, CD80, and CD86. Interestingly, it was also observed that despite enhancing the expression of such molecules, *L-braziliensis-*infected DCs produced higher levels of TNF-α in response to stimulation with LPS. These findings corroborate the hypothesis that uninfected and *Leishmania*-infected DCs can act conjointly, yet distinctly, to promote immune responses against the parasite, since uninfected DCs can lead to enhanced T cell activation, while the production of TNF-α by infected DCs may contribute to the control of parasite proliferation at the site of infection (81).

Paradoxically, DCs are also able to present exogenous antigens through MHC-I, with significant consequences on the activation of CD8 T cells (82). It has been reported that this phenomenon, also known as cross-presentation, is of great importance to the expansion of antigen-specific cytotoxic CD8 T cells, which are responsible for eliciting an effective immune responses against *Leishmania*. Accordingly, in this context, DC figure as the most potent inducers of IFN-γ production by CD8+ T lymphocytes. Brewig et al. demonstrated that, in experimental leishmaniasis, the priming of CD4^+^ and CD8^+^ T cell relies essentially on the activity of distinct DC subtypes (83). Indeed, it was reported that the depletion of Langerin^+^ DCs was associated with the reduced proliferation of *L. major*-specific CD8+ T cells. As a consequence, the amount of primed CD8 T cells found at the site of infection and in lymph nodes was significantly reduced (83). In a similar vein, a study conducted by Ashok et al revealed the importance of cross-priming DCs in the effective constraint of *L. major* infection. It was shown that Batf3^−/−^ mice (which lack CD8^+^/CD103^+^ DCs) exhibited increased susceptibility *to L. major* (84). Furthermore, a study by Lemos et al explored the function of CD8^+^ DCs in antigen presentation during *L. major* infection in a murine model that restricted the expression of MHC-II to CD8a^+^/CD11b^+^ DCs. Notably, it was observed that CD8a^+^/CD11b^+^ DCs could efficiently restrain *L. major* infection by eliciting the effective constraint of parasites by CD4 T cells (85).

Numerous studies have revealed that the underlying mechanisms of antigen processing depend not only on the constitutive proteasome or the immunoproteosome, but also on the involvement of alternative molecular machineries of cytosolic degradation, such as tripeptidyl peptidase II (TPPII) and nardilysin (86–88). TPPII is a known eukaryotic peptidase related to several cellular processes, such as antigen processing, apoptosis and cell division. However, it should be noted that TPPII activation occurs mainly when proteasome function becomes compromised (89).

Although little is known about the detailed mechanisms of cytosolic endopeptidases, such as Nardilysin, their role seems to be indispensable in the generation of some specific epitopes (90). The importance of alternative antigen processing machinery should be further investigated in the context of leishmaniasis.

Although the participation of CD8^+^ T cells in *Leishmania* infection is still controversial, growing evidence indicates that protective responses rely substantially on the effective dendritic cell-mediated activation of cytotoxic lymphocytes (86, 87, 91, 92).

## *Leishmania* Affects the Migration of DCS

Immature DCs strategically reside in peripheral tissues, where they exercise their main function as immune guards. As discussed previously, these sentinels specialize in antigen uptake via their apparatus to internalize foreign particles. In peripheral tissues, PAMP-mediated activation confers an immunostimulatory phenotype to DCs, characterized by the upregulation of molecules also associated with an enhanced migratory ability. Subsequently, DCs migrate toward lymph nodes, where they exchange information with naïve T cells via the antigen presentation process (93).

**Figure 2:**
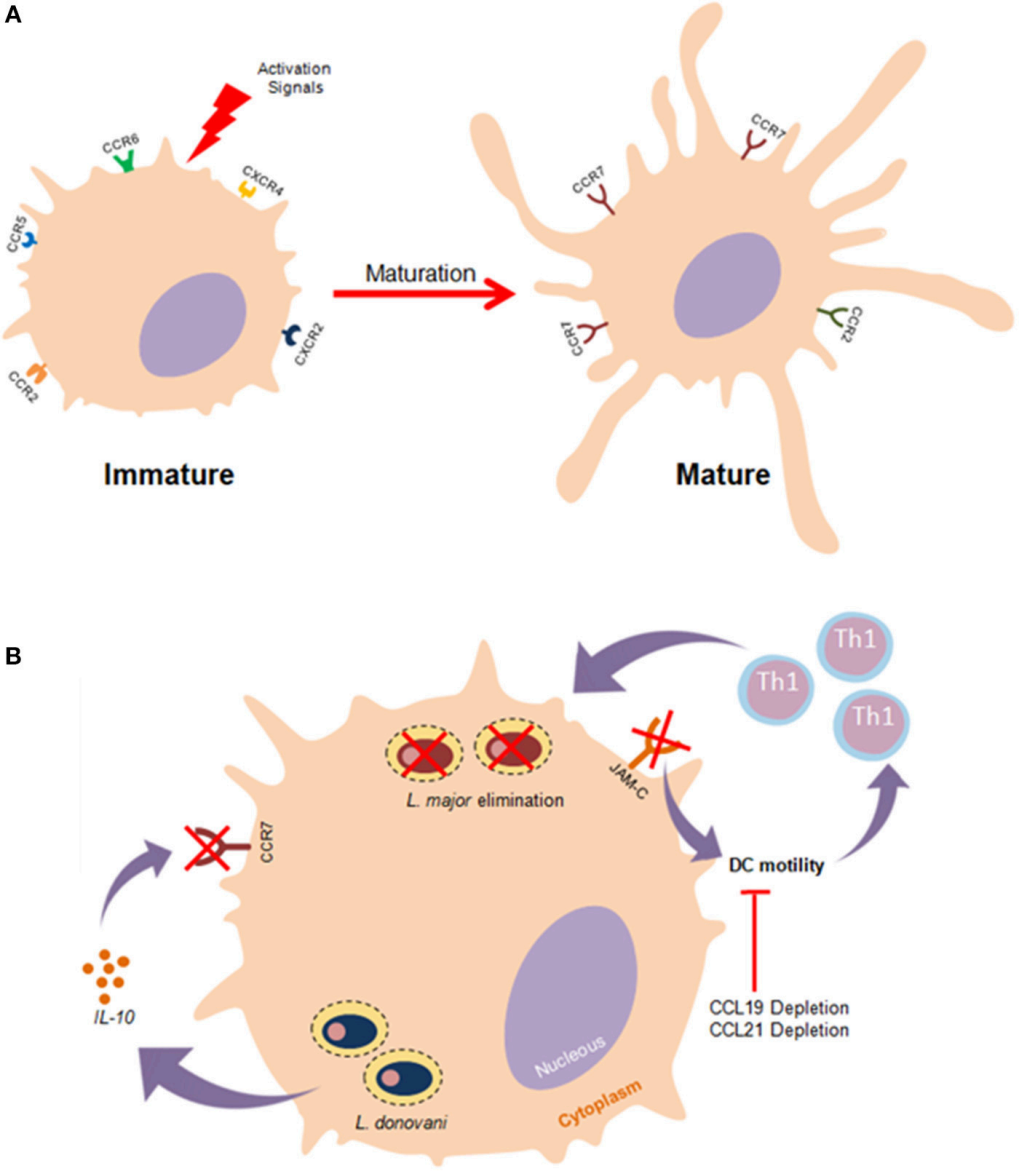
Effects of *Leishmania* parasites in DC migration. **(A)** Immature DCs exhibit particular membrane surface markers such as CCR2,CCR5,CXCR4, and CXCR2. Once stimulated by effective activation signals, DCs undergo a maturation process that culminate in the upregulation CCR7 expression as well as enhanced mobilility. **(B)** The establishment of effective immune responses against *Leishmania* depends substantially on the migration of DCs to lymph nodes where these cells activate T lymphocytes. *Leishmania* impairs the highly coordinated process of DC migration as an evasion strategy to prevent the leishmanicidal effects of the adaptative immunity. *L. donovani* mitigates the functionality of CCR7 in an IL-10 dependent manner, thus hampering DC migration. Furthermore, it has been documented that the experimental blockage of junctional adhesion molecule-C (JAM-C) enhances DC migration and immunity against *L. major*.

In order to ensure precise mobilization, the migration of DCs needs to be highly coordinated and regulated by particular recruitment signals (94). The primary mechanisms of DC migration involve the cooperative action of chemokines and their receptors. Chemokine receptors are typically transmembrane proteins associated with G-proteins whose activation triggers signaling pathways responsible for the promotion of cell mobilization (95). Some evidence seems to suggest that DC subtypes exhibit diverse chemokine receptors, conferring subtype-specific migration dynamics. Commonly, immature DCs express CCR2, CCR5, CCR6, CXCR2, and CXCR4 in a predominant fashion. Upon pathogen-mediated activation, DCs undergo a maturation processes that culminates in the crucial upregulation of CCR7 (Figure 2A) (96).

By way of evolution, protozoan parasites developed strategies to mitigate DC functioning by inhibiting access to T cells, thusly restricting the establishment of efficient adaptive immune responses. In the context of flagellate protozoan infection, successful DC migration to draining lymph nodes (dLNs) is substantially dependent on both CCR2 and CCR7 expression (97). Several studies have reported that *Leishmania* can induce a reduction in rates of DC migration (98, 99). *In vitro* studies have elucidated the roles of both soluble products and membrane constituents, such as *L. major* LPG, in the inhibition of DC motility (98, 100). The underlying molecular mechanisms of *Leishmania*-induced mitigation of DC motility remain elusive. A recent study suggested that *L. major* exploits the junction adhesion molecule C (JAM-C) to reduce DC migration rates, and demonstrated that the experimental blockage of this molecule enhanced the immunological response against this parasite (101). An investigation in an animal model exhibited an *L. major-* susceptible phenotype, suggesting that the depletion of CCR2 culminated in poor DC migration and a skewed Th2 immune response (102). Furthermore, infection with *L. donovani*, a viscerotropic species, promotes an inhibition in the expression of CCR7 mediated by IL-10 production, which ensures that DCs will not be able to reach splenic regions, thereby contributing to the progression of visceral leishmaniasis (Figure 2B) (103). Another *in vivo* study associated CCL19/CCL21 deficiency with a reduction in both DC mobility and resistance to *L. donovani* infection (103).

Collectively, the current data suggest that efficacious DC migration is essential to the establishment of effective responses against *Leishmania* parasites. The literature clearly indicates that these parasites employ a plenitude of strategies to prevent DCs from activating T cells during the course of several clinical forms of leishmaniasis. Deciphering the complex dynamics surrounding the *Leishmania*-mediated impairment of DC mobilization will provide new insights into the evasion mechanisms employed by these parasites and elucidate their effects on the immunopathogenesis of leishmaniasis.

## Metabolic Reprogramming During DC Activation

Faced with infection and inflammation, DCs must cope with increasing catabolic and anabolic demands via the redirection of a plethora of metabolic pathways to support their major immune functions (104). Typically, the metabolism of inactive DCs is characterized by the central roles of oxidative phosphorylation (OXPHOS) and fatty acid oxidation (FAO), for energy supply and biomolecule synthesis, respectively (105). New evidence suggests that, after the initiation of PAMP-mediated activation, DCs undergo metabolic reprogramming, relying substantially on anaerobic glycolysis for ATP production, a process characterized by the conversion of pyruvate into lactate. Despite being ineffective in the generation of ATP, glycolysis can be coupled with several anabolic pathways, such as fatty acid synthesis and the pentose phosphate pathway, allowing for the biosynthesis of other macromolecules, namely lipids and nucleotides, respectively (106, 107). In this scenario, DCs exhibit low rates of oxidative phosphorylation. These deviations in the metabolic repertoire of DCs are prominent regulators of immune responses, as metabolic enzymes and their products can influence the establishment of inflammation (108).

**Figure 3:**
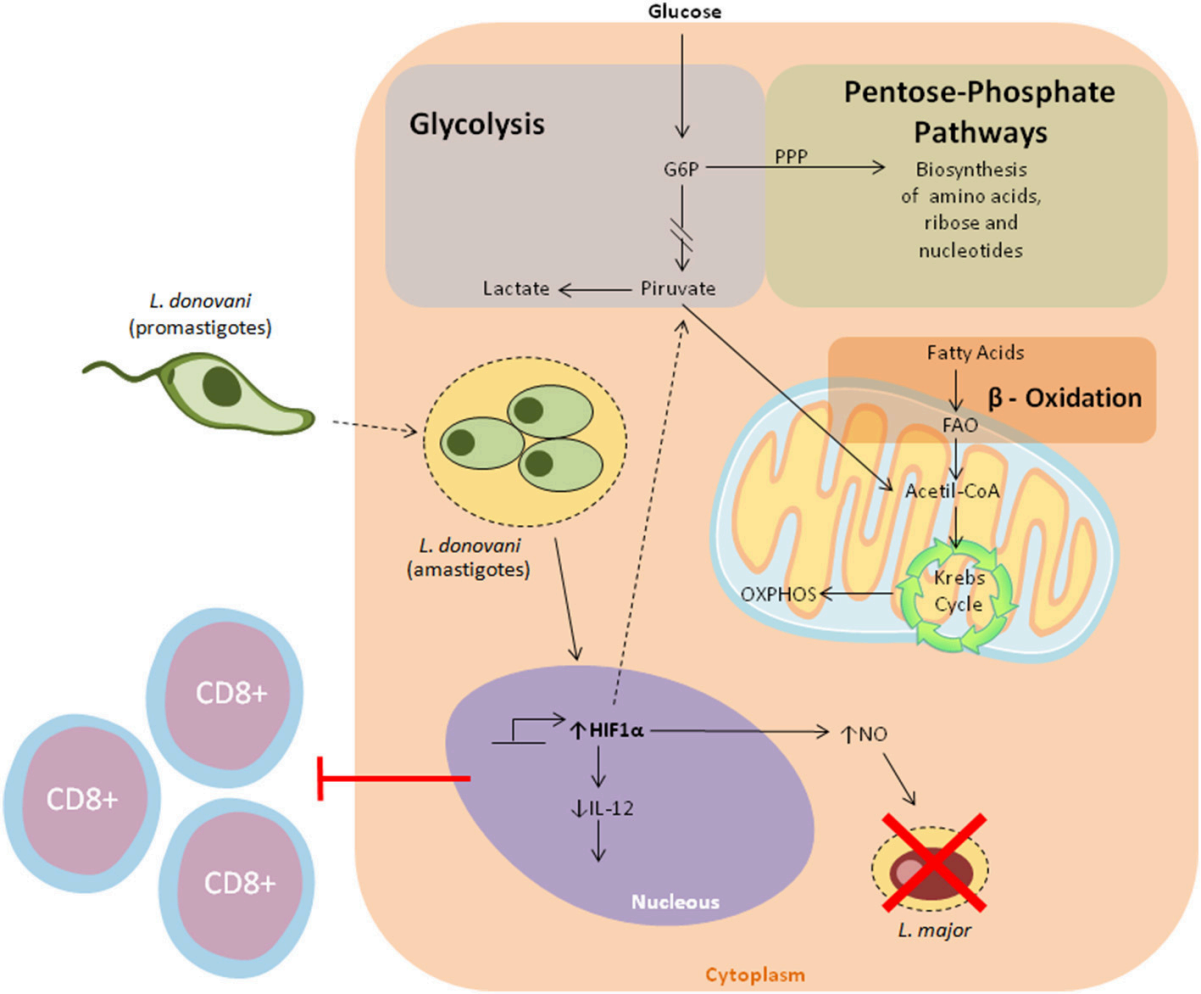
Overview of the major metabolic pathways in Dendritic cells. Cellular metabolic processes provide DCs with the necessary energy to sustain their immunological functions. In a resting state, DCs rely on oxidative phosphorylation to meet their bioenergetic requirements. When faced with pathogens, DCs undergo metabolic reprogramming skewed toward aerobic glycolysis for ATP generation, and the establishment of pro-inflammatory responses. Of note, different reports seem to suggest that HIF1a play a dual role in metabolic reprogramming in leishmaniasis. It has been shown that *L. donovani* infection enhances the stabilization of HIF1a, which in turn leads to decreased Il-12 production. Conversely, It has been demonstrated that HIF1a increases nitric oxide production, which subsequently results in *L. major* elimination.

In this inflammatory milieu, immune cells are poorly supplied with oxygen and nutrients for their metabolic processes, leading to the activation of hypoxia-inducible transcription factor 1α (HIF-1α) (109). Recently, HIF-1α was recognized as a major player in the induction of glycolysis, since it promotes the transcription of several enzymes involved in glucose metabolism (110). Nevertheless, its expression was shown to favor *L. donovani* infection in a model of chronic visceral leishmaniasis, as increased HIF-1a expression in murine splenic DCs was correlated with decreased IL-12 production, allowing parasite survival through limited Th1 cell expansion (111). In consonance with these observations, Hammani et al. demonstrated the importance of the IRF-5/HIF1α transcription factor axis in the impairment of DCs to promote the expansion of CD8^+^ T cells (112). Conversely, *in vitro* experiments showed that HIF1α enhanced both *Leishmania major* elimination and levels of NO production in macrophages (Figure 3) (113). Together, while these observations suggest that HIF1α downregulates some DC functions against *Leishmania*, this effect may be cell-specific. Moreover, a recent study highlighted the contribution of two energetic sensors, Sirtuin 1 (SIRT1) and AMP-activated protein kinase (AMPK), to *L. infantum* survival and replication in macrophages (114).

Given the relevance of the metabolic processes of DCs in supporting the immunobiological functioning of these cells, it is unsurprising that an increasing number of studies have contemplated this interesting topic in recent years. Nevertheless, few studies have attempted to investigate the role of DC immunometabolism in *Leishmania* infection. Currently, the molecular players involved in metabolic reprogramming and the mechanistic basis of immunometabolism continue to remain elusive in the context of leishmaniasis.

## Effects of Epigenetic Modifications on dc Development and Course of Infection

Interactions between host cells and parasites prompt several alterations in a range of biological processes occurring in DCs, including epigenetic alteration via modified gene expression. This phenomenon is not dependent on DNA sequence modifications and includes DNA methylation, histone modifications, chromatin remodeling and regulation by non-coding RNAs (115–117).

The activation of transcription factors is one of the major regulatory elements occurring in epigenetic alterations (118, 119). PU.1 transcription factor has been described as an essential TF for the development and functioning of DCs, as evidenced by the expression of FLT3, granulocyte-macrophage colony-stimulating factor receptor (GM-CSFR) and macrophage colony stimulating factor receptor (M-GSFR) (119, 120). It has been demonstrated that PU.1 is also involved in the regulation of basal expression of DC-SIGN, which in turn influences the repertoire of antigen uptake in DCs (121). PU.1 can also regulate the promoter region of genes CD80 and CD86 in murine bone marrow–derived DCs, leading to the overexpression of these co-stimulatory molecules, thereby enhancing DC migration and the activation of T cells (122).

In face of tissue damage or infection, several modifications in the histones alter chromatin conformation, leading to changes in the expression profile of critical genes in specific DC subsets (123, 124). Tserel et al. showed by GWAS (Genome-wide Association Study) that histone modifications can influence the processes of differentiation, phagocytosis and antigen presentation in moDCs through the upregulation of surface marker expression and chemokine production. Similar findings have been reported in macrophages, reinforcing the similarity of epigenetic mechanisms in the development of both cell types (125).

The importance of epigenetic changes in DCs infected by *Leishmania* remains unclear. However, *L. donovani* infection in macrophages was shown to lead to changes in the methylation of CpGs sites via parasite exosome secretion, which may enhance parasite replication and survival (126). Furthermore, *L. amazonensis* infection promoted epigenetic modifications at the IL-10 *locus* in murine macrophages, which activated ERK1/2 pathways and promoted parasite survival (127). In addition, this parasite species can upregulate histone deacetylases (HDACs), which enhances iNOS promoters in macrophages, thusly favoring infection (128).

Taken together, in addition to playing a crucial role in the development of DCs, these findings seem to suggest that interactions between *Leishmania* and immune cells can trigger epigenetic modifications that may alter the course of the infection. However, much remains to be elucidated with regard to this topic.

## Conclusion Remarks

DCs are relevant immunological agents in the concatenation of innate and adaptative branches of immunity. Here, we have attempted to integrate recent advances in molecular aspects of the immunobiological functioning of DCs with the current state of understanding regarding the pathogenic mechanisms of leishmaniasis. Although a large body of evidence supports the central role of DC activation in the establishment of responses against *Leishmania* parasites, the overwhelming complexity of *Leishmania*-DC interactions impedes the attainment of a comprehensive understanding of the molecular processes involved in DC activation. Further clarification is required to unravel the interplay between different DC subtypes and different species and life cycle stages of *Leishmania* as well as how parasites subvert particular aspects of DC activation in the effort to successfully establish infection. Finally, an enhanced understanding of the fundamental molecular events underlying DC activation will lead to the expansion of our current base of knowledge surrounding leishmaniasis as well as offer new therapeutic targets.

## Author Contributions

RT and SN designed the review and wrote the manuscript. IN, MR, IS, and RL assembled the review and wrote the manuscript. NM and CB supervised the work, designed the review, and wrote the manuscript.

### Conflict of Interest Statement

The authors declare that the research was conducted in the absence of any commercial or financial relationships that could be construed as a potential conflict of interest.
